# The effect of COVID-19 on malaria cases in Zambia: a mixed effect multilevel analysis

**DOI:** 10.1186/s12936-024-04882-6

**Published:** 2024-03-22

**Authors:** Mutale Sampa, Ronald Fisa, Chilombo Mukuma, Mercy Mwanza, Busiku Hamainza, Patrick Musonda

**Affiliations:** 1 School of Public Health, Department of Epidemiology and Biostatistics, The University of Zambia, University of Zambia, Lusaka, Zambia; 2 Southern African Institute for Collaborative Research and Innovation Organization (SAICRIO), Lusaka, Zambia; 3 National Malaria Elimination Centre, Ministry of Health, Lusaka, Zambia

**Keywords:** COVID-19, Malaria, Mixed effects, Poisson, Sub-Saharan Africa

## Abstract

**Background:**

The burden of Malaria in Zambia remains a challenge, with the entire population at risk of contracting this infectious disease. Despite concerted efforts by African countries, including Zambia, to implement malaria policies and strategies aimed at reducing case incidence, the region faces significant hurdles, especially with emerging pandemics such as COVID-19. The efforts to control malaria were impacted by the constraints imposed to curb its transmission during the COVID-19 pandemic. The aim of the study was to assess the effect of the COVID-19 pandemic on malaria cases in Zambia and the factors associated by comparing the COVID-19 period and the pre-COVID-19 era.

**Methods:**

This was a cross-sectional panel study in which routinely collected programmatic data on malaria was used. The data were extracted from the Health Management Information System (HMIS) for the period January 2018 to January 2022. The period 2018 to 2022 was selected purely due to the availability of data and to avoid the problem of extrapolating too far away from the period of interest of the study. A summary of descriptive statistics was performed in which the number of cases were stratified by province, age group, and malaria cases. The association of these variables with the COVID-19 era was checked using the Wilcoxon rank-sum test and Kruskal‒Wallis test as applicable. In establishing the factors associated with the number of malaria cases, a mixed-effect multilevel model using the Poisson random intercept and random slope of the COVID-19 panel. The model was employed to deal with the possible correlation of the number of cases in the non-COVID-19 panel and the expected correlation of the number of cases in the COVID-19 panel.

**Results:**

A total of 18,216 records were extracted from HMIS from January 2018 to January 2022. Stratifying this by the COVID-19 period/era, it was established that 8,852 malaria cases were recorded in the non-COVID-19 period, whereas 9,364 cases were recorded in the COVID-19 era. Most of the people with malaria were above the age of 15 years. Furthermore, the study found a significant increase in the relative incidence of the COVID-19 panel period compared to the non-COVID-19 panel period of 1.32, 95% CI (1.18, 1.48, p < 0.0001). The observed numbers, as well as the incident rate ratio, align with the hypothesis of this study, indicating an elevated incidence rate ratio of malaria during the COVID-19 period.

**Conclusion:**

This study found that there was an increase in confirmed malaria cases during the COVID-19 period compared to the non-COVID-19 period. The study also found Age, Province, and COVID-19 period to be significantly associated with malaria cases.

## Background

Globally, malaria is still the most important parasitic disease and is responsible for a quarter of all deaths among children under 5 years in sub-Saharan Africa (SSA) [1]. Efforts for global malaria control and elimination have achieved large successes during the last two decades, but progress has stalled in recent years, and the COVID-19 pandemic could largely reverse the overall trend [2]. Malaria control largely depends on the mass distribution of long-lasting insecticidal nets (LLINs), seasonal malaria chemoprevention (SMC), and indoor residual spraying of insecticide (IRS) across communities and households. With a slide-based diagnosis, rapid diagnostic tests (RDTs), case management delivered through trained health staff, and increasing awareness, the malaria burden has been significantly reduced over the years [3, 4].

The World Health Organization (WHO) officially declared the COVID-19 pandemic a public health emergency of international concern in January 2020. As of September 2023, the WHO [5] reported that there have been over 700 million (exactly reported figure was 770,778,396) confirmed cases of COVID-19, of which approximately 7 million died from the disease. The burden of malaria in Zambia remains a challenge, with the entire population at risk of contracting this infectious disease. Despite concerted efforts by African countries, including Zambia, to implement malaria policies and strategies aimed at reducing case incidence, the region faces significant hurdles. Africa, with its weak public health infrastructure and limited resources, is already grappling with the highest infectious disease burden and chronic non-communicable diseases. The fight against malaria is further complicated by constrained health budgets, shortages of essential medicines and personal protective equipment, and a shortage of personnel trained in critical care [6, 7].

The COVID-19 pandemic worsened the existing health challenges in low- and middle-income countries, particularly in Africa [8]. Initial predictions anticipated that Africa would be severely impacted due to its weak health systems, prevailing poverty, and the high burden of other infectious diseases [9]. The pandemic brought unprecedented challenges to health systems worldwide, diverting attention and resources away from controlling non-COVID-19 diseases like malaria. Zambia experienced significant strain on its healthcare systems during the peak of the COVID-19 pandemic. The surge in demand for hospital services outpaced the available facilities, resulting in system overload. The efforts to control malaria were impacted by the constraints imposed during the COVID-19 pandemic to curb its transmission. Measures like social distancing prompted the temporary suspension of interventions, such as indoor residual spraying. Although mosquito nets continued to be distributed at health facilities, individuals minimized their visits to these facilities unless they displayed symptoms indicative of COVID-19.

The study hypothesized that the incidence of malaria cases and fatalities in Zambia might have risen during the COVID-19 period, attributing this potential increase to the direct and indirect consequences of the pandemic in malaria-endemic regions, especially in Sub-Saharan Africa (SSA) [5]. Zambia relies heavily on external funding for disease-specific initiatives in its healthcare systems [9]. Consequently, efforts aimed at managing the COVID-19 crisis might have influenced the capacity to address ongoing endemic health challenges [3, 10].

Amidst these challenges, our investigation sought to assess the effect of the COVID-19 pandemic on malaria cases in Zambia by comparing the COVID-19 period and the pre-COVID-19 era. The study used routinely collected data by Ministry of Health (MoH) in the health information system (HMIS) to determine factors associated with malaria cases during the period. The choice to use routine data harnessed the pre-existing information gathered by the MoH from diverse health facilities. This decision provided the chance to analyze data for a wide range of health facilities as well as data pertaining to health facilities that are challenging to access.

## Methods

This was a cross-sectional study using routinely collected programmatic data on malaria cases. The data were extracted from the HMIS with permission from the Zambia MoH from 2018 to January 2022. A variable period was generated by dividing the data into two panels: the COVID-19 period and the non-COVID-19 period. The COVID-19 period was defined as the period from March 2020 to January 2022. Furthermore, the non-COVID-19 period was defined from January 2018 to February 2020. The reasoning behind this categorisation was to do with the view that the first case of COVID-19 in Zambia was diagnosed in March 2020, and the data made available were from January 2018 to January 2022. The outcome variable was the number of confirmed malaria cases per month over the years, which was a count, and the explanatory variables included were age, year (2018, 2019, 2020, 2021, and 2022), period (COVID-19 period, and non-COVID-19 period), and province. Data collected by MoH in the Health Information System HMIS has limited variables; the data is aggregated and, therefore, lacks individual-level variables. Hence, a few explanatory variables were used in this study.

### Statistical analysis

All analyses were performed using STATA^®^ version 17 (StataCorp, College Station, TX, USA) software. Malaria cases and positivity rates were described and presented using tables and graphs. To test for associations between the variable period and malaria cases, we used the Wilcoxon rank-sum test, a nonparametric equivalent of Student's t-test, and the Kruskal‒Wallis test, a nonparametric equivalent of ANOVA. Non-parametric tests were used because malaria cases violated the normality assumption.

For inferential statistics, due to the possible correlation between cases seen in the non-COVID-19 period panel and those seen in the COVID-19 period panel, the study used the Poisson multilevel mixed model of random intercept and random slope of the COVID-19 panel with unstructured working correlation. This model took into account the correlation within and between the COVID-19 panel and the variables present. Mathematically, the model used is shown below:

$$\begin{gathered} \mu_{ij} \equiv E(y_{ij} \left| {{\mathbf{x}}_{ij} } \right.) = \exp \{ \beta_{1} + \beta_{2} x_{2i} + {{\varvec{\upbeta}}}_{3ij} {\mathbf{x}}_{3ij} + {{\varvec{\upbeta}}}_{4ij} {\mathbf{x}}_{4ij} + \zeta_{1j} + \zeta_{2j} x_{2i} \} \hfill \\ \, = \exp \{ (\beta_{1} + \zeta_{1j} ) + (\beta_{2} + \zeta_{2j} )x_{2i} + {{\varvec{\upbeta}}}_{3ij} {\mathbf{x}}_{3ij} + {{\varvec{\upbeta}}}_{4ij} {\mathbf{x}}_{4ij} \} \hfill \\ \end{gathered}$$

where $\mu_{ij}$ is expected incident rate ratio of malaria cases, $y_{ij}$ is the number of confirmed malaria cases for the observation period covering the NON-COVID-19 period to COVID-19 period $i$, for the $j{\text{th}}$ malaria count per month over the years, ${\mathbf{x}}_{ij}$ is a vector of covariates, namely, COVID-19 panel covariate ($x_{2ij}$), a vector of covariates associated with age categories (${\mathbf{x}}_{3ij}$, i.e., < 1 year old, 1–4 years old, 5–14 years old, ≥ 15 years old), a vector of covariates associated with Province categories (${\mathbf{x}}_{4ij}$, i.e., Southern, Central, Copperbelt, Eastern, Luapula, Lusaka, Muchinga, Northern, North Western, Western), $\beta_{1}$ is the intercept, $\zeta_{1j}$ is the random intercept, $\beta_{2}$ is the incident rate ratio for COVID-19 relative to non-COVID-19 period, ${{\varvec{\upbeta}}}_{3ij}$ is a vector of regression coefficients (incident rate-ratios) of age categories relative to age category < 1 year old, ${{\varvec{\upbeta}}}_{4ij}$ is the vector of regression coefficients (incident rate-ratios) of Provinces relative to Southern Province and finally $\zeta_{2j}$ is the random coefficient associated with the regression coefficient $\beta_{2}$.

## Results

During the non-COVID-19 period, a total of 8852 records were submitted to the MOH, and a total of 9364 records were submitted during the COVID-19 period. The median number of confirmed malaria cases was 485, and the interquartile range (IQR) was 110 to 1518 confirmed malaria cases in the non-COVID-19 period. The corresponding confirmed median number of confirmed malaria cases in the COVID-19 period was 547 (IQR = 143–1576) (see Table 1). The difference in the median number of confirmed cases during the non-COVID-19 period to the COVID-19 period was statistically significant (p = 0.0001, see Table 1) in that there were more confirmed malaria cases in the COVID-19 period. Table 1 also shows the median distribution of confirmed malaria cases over age groups and across provinces stratified by non-COVID-19 period and COVID-19 period. The median numbers across all categories indicate that the median number of confirmed malaria cases was higher in the COVID-19 period than in the non-COVID-19 period.

**Table 1:** Median confirmed malaria cases stratified by non-COVID-19 period to those of COVID-19 period

Factors	COVID-19		p value
	Non-COVID period (N = 8852)	COVID-19 period (N = 9364)	
Confirmed malaria cases median (IQR)	485 (110, 1518)	547 (143, 1576)	0.0001^W^
Age group-median (IQR)			
< 1 year old	147 (38, 347)	160 (43, 372)	0.0001^K^
1–4 years old	645 (168, 1367)	683 (200, 1453)	
5–14 years old	234 (165, 234)	839 (244, 1804)	
≥ 15 years old	1635 (511, 3486)	2208 (800, 4701)	
Province-median (IQR)			
Southern	20 (5, 67)	26 (7, 97)	0.0001^K^
Central	409 (78, 1378)	427 (124, 1322)	
Copperbelt	1119 (365, 3037)	1284 (429, 2812)	
Eastern	751 (235, 2108)	610 (191, 1661)	
Luapula	1138 (493, 2147)	1197 (523, 2374)	
Lusaka	96 (28, 258)	142 (41, 389)	
Muchinga	847 (313, 1814)	903 (336, 1922)	
Northern	787 (309, 1599)	881 (425, 787)	
North Western	957 (353, 2371)	1039 (408, 2371)	
Western	191 (46, 735)	403 (97, 1127)	

*W* Wilcoxon rank-sum test, *K* Kruskal‒Wallis test

**Fig. 1:**
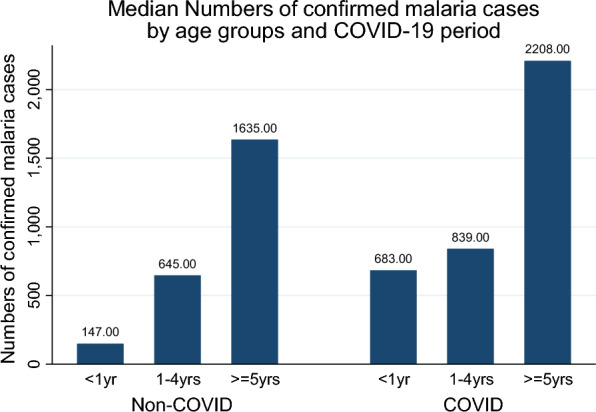
Median numbers of confirmed malaria cases

Figure 1 shows the bar graph substantiating the differences in median confirmed malaria cases for the non-COVID-19 period with those of the COVID-19 period.

**Fig. 2:**
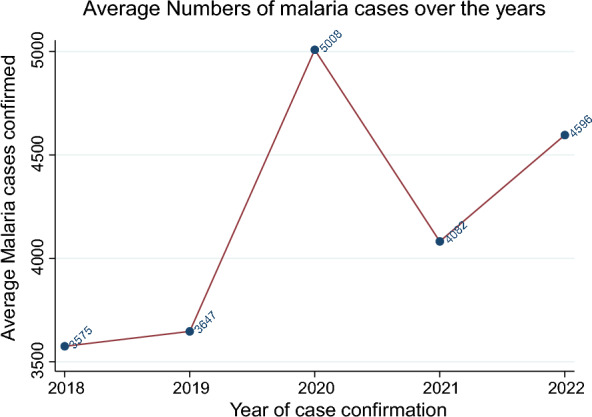
Average confirmed malaria cases from 2018 to 2022

Figure 2 shows the distribution of average confirmed cases over the study period. There is a sharp rise in confirmed average malaria cases in 2020, with a fall in 2021 followed by a rise in 2022. We only had one month of data in 2022, yet the confirmed average number of malaria cases was higher than that in the 2018, 2019 and 2020 periods. These findings also suggest that there were many more malaria cases in the COVID-19 period.

Descriptive analyses such as those shown in Table 1, Figs. 1 and 2 are not telling the whole story as they are looking at the information in isolation from other explanatory factors. To fully appreciate what is going on, data was analyzed further by also controlling with other explanatory variables. The other issue is that the analysis performed in Table 1, Figs. 1, and 2 assumes data independence over the study period. However, given the catastrophic event of the COVID-19 period panel, one would expect some correlation within the panel of non-COVID periods and another correlation in the COVID-19 panel period [11, 12]. To avoid bias in the estimates, the analysis needed to take this dependence into account.

Therefore, to take the dependence into account, a Poisson mixed multilevel model was used, where the random part is the intercept and the slope of the panel variable and the other variables were kept fixed. The model also implicitly took into account overdispersion in the count outcome [13].

Table 2 shows the results from the random intercept and slope Poisson model as described above, taking into account the correlation within the panels. The main result to interpret is the a priori variable, which is the panel variable (COVID-19 variable). We see that the relative incidence of the COVID-19 panel period compared to the non-COVID-19 panel period is 1.32, 95% CI (1.18, 1.48, p < 0.0001).

**Table 2:** Mixed effect estimates of the incidence rate ratio of the number of cases of malaria using Poisson random intercept and random slope of the COVID-19 panel

Factors	Estimates		
	IRR	p value	95% (CI)
Fixed part			
Age groups			
< 1 year old	Reference	n/a	n/a
1–4 years	3.96	< 0.0001	(3.95, 3.97)
5–14 years old	4.98	< 0.0001	(4.97, 4.99)
≥ 15 years old	6.37	< 0.0001	(6.35, 6.38)
Province			
Southern#	Reference	n/a	n/a
Central	11.4	< 0.0001	(6.61, 19.7)
Copperbelt	30.9	< 0.0001	(17.4, 54.4)
Eastern	15.4	< 0.0001	(8.77, 27.0)
Luapula	23.5	< 0.0001	(13.2, 41.6)
Lusaka	4.06	< 0.0001	(2.13, 7.73)
Muchinga	19.2	< 0.0001	(10.3, 35.5)
Northern	18.9	< 0.0001	(10.9, 32.8)
Northwestern	25.0	< 0.0001	(14.2, 43.8)
Western	12.7	< 0.0001	(7.84, 20.4)
Random part			
COVID-19*			
2018 to Feb2020 (not COVID-19)	Reference	n/a	n/a
March2020 to Jan2022 (yes COVID-19)	1.32	< 0.0001	(1.18, 1.48)
Standard deviation COVID-19	0.618		(0.542, 0.703)
Standard deviation (Intercept)	0.937		(0.806, 1.090)
Correlation (COVID-19, Intercept)	−0.689		

^#^For Province, we have used Southern Province because it had the lowest incidence of malaria cases on average

^*^Not COVID-19 period is defined as the period from January 2018 to February 2020, and the COVID-19 period is from March 2020 to January 2022 (given that the first COVID-19 case in Zambia was diagnosed in March 2020)

The estimates suggest that for each year of COVID-19, the incidence rate ratio of malaria cases varied by an approximate increase of approximately 32% with a 95% confidence interval of approximately 18–48% increase when controlling for age groups and the province where the data came from.

The standard deviation of the incident rate ratio of the COVID-19 panel was approximately 0.618 and that of the intercept was approximately 0.937. Overall, there was a negative correlation between the COVID-19 panel and an intercept of approximately 0.689. This suggests that during the COVID-19 years panel, as the number of years increased from 2020 to 2022, the incidence rate ratio (IRR) of the number of malaria cases decreased; however, relatively speaking, the IRR was still higher during the COVID-19 period than during the non-COVID-19 period.

## Discussion

The COVID-19 pandemic was an unprecedented event around the world. This generation saw the whole world being turned upside down. Countries around the world implemented lockdowns in which all citizens were asked to be quarantined either in their homes or in some institutions. Entertainment events were cancelled, and sports events were also cancelled. This period brought in a unique set of circumstances in how human beings behaved. Human beings behave nothing like they have done before for over a century. In Zambia, this pandemic affected the distribution and resource allocation to diseases such as malaria and HIV. At national level, the MoH decided to use most of the wards for Covid patients and most of the malaria patients were discharged and advised to go home. This was all due to limited wards for patients coupled with the nature of the disease (COVID-19) and its transmission.

In addition to different behaviours being forced on human beings around the world, health systems were pushed to their limit, let alone those in low-middle-income countries such as Zambia. Globally, it was widely thought that Africa was not going to cope well with the COVID-19 pandemic.

The main setbacks in health system preparation included the lack of available health services needed for the pandemic, inadequate resources and equipment, and limited testing ability and surge capacity for COVID-19. Reduced flow of patients and missing scheduled appointments were among the most common impacts of the COVID-19 pandemic in Zambia. Furthermore, there was no availability of telephone consultations, repurposing of available services, establishment of isolation centres, or provisions of COVID-19 guidelines in some settings.

This study analyzed routinely collected data from the MoH in Zambia. Given the shake-up the world and indeed Zambia experienced during the COVID-19 pandemic, the study hypothesized that a number of existing diseases in Zambia, such as malaria, were likely to be neglected during the COVID-19 period compared to the non-COVID-19 period. This neglect was likely to manifest itself in an increasing number of cases of some diseases. In particular, the study hypothesized that the number of confirmed malaria cases would likely increase during the COVID-19 period compared to the non-COVID-19 period. In this regard, as shown in Table 1, the increase in median confirmed malaria cases during the COVID-19 period (547 cases) compared to the non-COVID-19 period (485 cases) suggests that malaria was not prioritized as it should have been. Further, an increasing trend in malaria cases was observed in all provinces except in Eastern province where there was a decrease from 751 cases in the non-Covid period to 610 cases in the COVID period. This is an indication that lack of resources by the ministry led to an increase in malaria cases in 9 provinces of the country. This is consistent with findings obtained from a projected by the WHO (2020) in which 75% decrease in insecticide-treated nets (ITN) distribution coupled with a 75% decrease in access to artemisinin-based combination therapy (ACT) was predicted to result in a 22% increase in malaria cases and doubling of malaria deaths within a year to 769,000 [14].

At the time of data extraction, since secondary data was used, there was a limitation in terms of variables to use, and age groups, provinces, and years were included. The study investigated which age group was most affected. COVID-19 affected the country differently from province to province. Further, the study also wanted to investigate how malaria cases were affected in different provinces of the country.

For the purposes of this study, the COVID-19 period was defined as the period from March 2020 to January 2022. Furthermore, the non-COVID-19 period was defined from January 2018 to February 2020. The reasoning behind this categorization was to do with the view that the first case of COVID-19 in Zambia was diagnosed in March 2020, and the data made available were from January 2018 to January 2022.

There is no study that has investigated confirmed malaria cases using a COVID-19 panel period as investigated in this paper. The rigorous analysis of the data suggests that indeed, there was an increase in confirmed malaria cases in Zambia as measured by the median number of malaria cases and the incident rate ratio. The routinely collected data were limited to only having information on age groups, provinces, and years. The study found that in nearly all of these variables, there was an increase in the number of median confirmed malaria cases in the COVID-19 period compared to the non-COVID-19 period.

While this is a unique study, it is sufficed to mention that in limited resource settings such as Zambia handling pandemics such as COVID-19 is usually a challenge. All emergencies and disasters in the country are supported by the disaster management and mitigation unit. This is not enough especially in times of pandemics, such as COVID-19. Lack of infrastructure is one of the reasons patients were moved from hospitals to home to accommodate COVID-19 patients hence there is need to build more infrastructure. In addition to lack of infrastructure, supply of malaria prevention kits such as mosquito nets are adversely affected. In future, it would be good for the government through MOH to set aside enough resources for such emergencies/pandemics.

## Limitations

This study suffers from the usual imprecise information coming from routinely collected data or the observational nature of the data, such as missing variables and/or missing data. Routine errors of how information is gathered may luck strictness of research conditions. Hence, the findings can only be considered hypothesis-generating, and generalizability is limited. However, in this study data from all the provinces of the country was used which gives a good representation of the country and a rigorous analytical method was employed that can take into account unobserved heterogeneity.

## Conclusion

This study shows that there was an increase in confirmed malaria cases during the COVID-19 period compared to the non-COVID-19 period. Using a mixed-effects Poisson model, this study revealed a statistically significant increase in malaria cases during the COVID-19 period, indicating that there was negligence in the malaria programmes and prevention strategies during the COVID-19 period. These measures include distribution of mosquito nets, indoor residual spraying, continuous surveillance and applying larvicides to kill mosquito larvae in waterlogged areas. The authors therefore recommend that the MOH Zambia and their cooperating partners such as global fund to pay particular attention to resetting the button in monitoring other diseases, such as malaria, which may have suffered a lack of attention due to COVID-19.

This study has further shown that this increase is significant hence this entails that the same could be the case for other diseases, including HIV, tuberculosis and cancer. Future research should focus on longitudinal studies to investigate the changes in number of cases of diseases during a pandemic. The study has indeed demonstrated that at the onset of COVID-19, the other diseases such as malaria were neglected as seen by increase in number of cases in the COVID-19 period compared to non-COVID-19 period.

Finally, this work has demonstrated that during a pandemic, it is highly likely that other diseases may be affected, and this has led to an increase in these diseases which in turn lead to death if well investigated. This is an eye opener regarding disease management..

## Data Availability

The data that support the findings of this study are available from the Zambian Ministry of Health, but restrictions apply to the availability of these data, which were used under license for the current study and so are not publicly available. Data are, however, available from the authors upon reasonable request and with permission of the Zambian Ministry of Health.

## Abbreviations

ZMIS: Zambia malaria indicator survey
HIV: Human immunodeficiency virus
HMIS: Health management information system
SSA: Sub-Saharan Africa
WHO: World Health Organization
